# *Strongyloides* species exhibit distinct behaviors on the skin of different mammals

**DOI:** 10.1186/s12879-025-11543-9

**Published:** 2025-10-03

**Authors:** Courtney R. Abell, Ruhi Patel, Elissa A. Hallem

**Affiliations:** 1 Department of Microbiology, Immunology, and Molecular Genetics, University of California, Los Angeles, Los Angeles, CA 90095 USA; 2 Environmental and Molecular Toxicology Interdepartmental PhD Program, University of California, Los Angeles, Los Angeles, CA 90095 USA; 3 Present Address: Department of Biological Sciences, Delaware State University, Dover, DE 19901 USA; 4 Molecular Biology Institute, University of California, Los Angeles, Los Angeles, CA 90095 USA

**Keywords:** *Strongyloides stercoralis*, *Strongyloides ratti*, Skin-penetrating nematode, Skin-penetration behavior, Host skin, Skin invasion

## Abstract

**Background:**

Skin-penetrating nematodes, including the human parasite *Strongyloides stercoralis* and the rat parasite *Strongyloides ratti*, are gastrointestinal parasites that infect when infective third-stage larvae (iL3s) invade host skin. To enter the skin, iL3s engage in skin-penetration behavior, in which they repeatedly push and puncture the skin surface before ultimately burrowing into the skin. These parasites have narrow host ranges, but the extent to which skin-penetration behavior contributes to host specificity remains unclear. Here, we examine the behavior of *S.* *stercoralis* and *S.* *ratti* iL3s on skin from three different closely related rodents – rat, mouse, and gerbil – as well as the behavior of *S.* *stercoralis* iL3s on human skin to better understand how behavior differs on host vs. non-host skin.

**Methods:**

We used population and single-worm ex vivo skin-penetration assays to compare the behaviors of *S.* *stercoralis* and *S.* *ratti* iL3s on different skin types.

**Results:**

We find that iL3s engage in repeated behavioral cycles on both host and non-host skin. However, these cycles vary across nematode species and skin types. *S.* *ratti* iL3s show similar behaviors on rat and mouse skin, although penetration takes longer to occur on mouse skin. In contrast, these iL3s show reduced and delayed skin-penetration behavior, and abort more skin-penetration attempts, on gerbil skin than on rat or mouse skin. *S.* *stercoralis* iL3s exhibit skin-penetration behavior at different frequencies on rat, mouse, gerbil, and human skin. These iL3s show increased skin-penetration behavior on gerbil skin relative to rat or mouse skin but show an even greater increase in behavior on human skin. Finally, we did not observe differences in iL3 behavior related to the age, sex, or genetic background of the host skin donor, suggesting that skin penetration is robust to differences in these host factors.

**Conclusions:**

Our results indicate that iL3s behave differently on different skin types and preferentially exhibit skin-penetration behavior on host skin or non-host skin from closely related mammals. Thus, enhanced skin-penetration behavior on host skin likely contributes to host selectivity.

**Supplementary Information:**

The online version contains supplementary material available at 10.1186/s12879-025-11543-9.

## Background

*Strongyloides stercoralis*, the causative agent of strongyloidiasis, is a skin-penetrating, gastrointestinal parasitic nematode that is a major source of disease in tropical and subtropical regions with poor sanitation infrastructure [1]. Obtaining accurate estimates of the prevalence of *S. stercoralis* infection is notoriously challenging due to the non-specific nature of strongyloidiasis symptoms, the ability of the parasite to maintain a low-level chronic infection, and the poor specificity and sensitivity of commonly used diagnostic tests [2–4]. While studies from the 1980s and 1990s suggested that 30–100 million individuals were infected with *S.* *stercoralis* [5, 6], this was widely believed to be an underestimate [7, 8], and a more recent study estimated that approximately 600 million individuals were infected with *S.* *stercoralis* in 2017 [1]. Infection with *S.* *stercoralis* can cause chronic gastrointestinal discomfort, eosinophilia, anemia, meningitis, and death in immunocompromised individuals [9]. While a limited number of anthelmintic drugs can be used to clear an existing infection, these drugs do not prevent re-infection and drug resistance is considered an imminent threat [9–11]. Thus, new strategies for preventing nematode infections are urgently needed. As an early step of the parasite-host interaction, skin penetration represents a potentially powerful target for anthelmintic intervention [12, 13]. A better understanding of skin penetration could facilitate the development of topical compounds that prevent infections by blocking skin invasion.

*S. stercoralis* infects hosts as infective third-stage larvae (iL3s). The iL3s inhabit the soil and migrate toward potential hosts using a variety of sensory cues. iL3s are repelled by carbon dioxide (CO_2_), which likely drives them away from the high-CO_2_ environment of host feces and into the surrounding soil environment to initiate host seeking [14–16]. iL3s are also attracted to host-emitted odorants and body heat, which likely directs them toward potential hosts [16–20]. A comparison of the olfactory behaviors of different species of skin-penetrating nematodes revealed that the olfactory preferences of iL3s reflect their host specificities rather than their genetic relatedness, suggesting that iL3s may distinguish hosts from non-hosts, and selectively navigate toward hosts, using olfactory cues [16, 20].

Once iL3s encounter a host, they invade the host by penetrating head-first through the skin [21, 22]. We recently showed that upon contact with host skin, iL3s engage in repeated behavioral cycles in which they crawl on the skin surface, push against the skin with their head in an apparent sampling of the surface, and puncture the surface with their head to attempt penetration [13]. While some penetration events are followed by the successful completion of skin penetration, in which the entire body of the iL3 enters the skin, other penetration attempts are aborted; in these cases, the iL3 either reverses out of the skin or makes a U-turn within the skin and then returns to the surface, crawls to a new location, and then resumes skin-penetration behavior. These behavioral cycles are driven by dopamine signaling – disrupting dopamine signaling pharmacologically or genetically, or chemogenetically silencing the dopaminergic neurons, severely impairs skin-penetration behavior [13].

Like other skin-penetrating nematodes, *Strongyloides* species have narrow host ranges. *S.* *stercoralis* can infect humans, some non-human primates, dogs, and possibly cats [23–32]. Recent comparisons of *S.* *stercoralis* strains isolated from humans and dogs revealed that some strains of *S.* *stercoralis* are more commonly found in both humans and dogs, while others are more commonly found only in dogs [23, 33–35]. However, the factors that contribute to differences in host preference between different strains of *S. stercoralis* remain poorly understood. Unlike *S.* *stercoralis*, the closely related species *Strongyloides ratti* has a natural host range that is limited to rats [36]. Although iL3s can penetrate both host and non-host skin [13], at least some iL3s appear to be more successful at penetrating host skin than skin from distantly related mammals. For example, *S.* *ratti* iL3s are more successful at penetrating rat skin than cat, dog, or bird skin [37]. In addition, a comparison of the behaviors of *S.* *stercoralis* iL3s on rat vs. human skin revealed that *S.* *stercoralis* iL3s attempt penetration more often on human skin than rat skin, whereas *S.* *ratti* iL3s attempt penetration more often on rat skin than human skin [13]. However, whether iL3s exhibit differences in skin-penetration behavior on the skin of closely related mammals, such as different rodent species, remained unclear.

Here, we examine the behaviors of *S.* *ratti* and *S.* *stercoralis* iL3s on skin from three rodent species: rat, gerbil, and mouse. We show that both *S.* *ratti* and *S.* *stercoralis* iL3s exhibit repeated cycles of skin-penetration behavior on skin from all three rodents and are capable of penetrating the skin of all three rodents; thus, skin-penetration behavior is largely conserved across different skin types. However, the frequency and timing of these behaviors varies across the different skin types such that iL3s show increased skin-penetration behavior on host skin or skin from closely related mammalian species. Our results highlight an important role for skin penetration in mediating host selectivity.

## Methods

### Ethics statement

All protocols involving vertebrate animals were approved by the UCLA Office of Animal Research Oversight (Protocol ARC-2011–060), which follows the standards of the AAALAC and the *Guide for the Care and Use of Laboratory Animals*.

### *S. ratti* and *S. stercoralis* strains

**Fig. 1:**
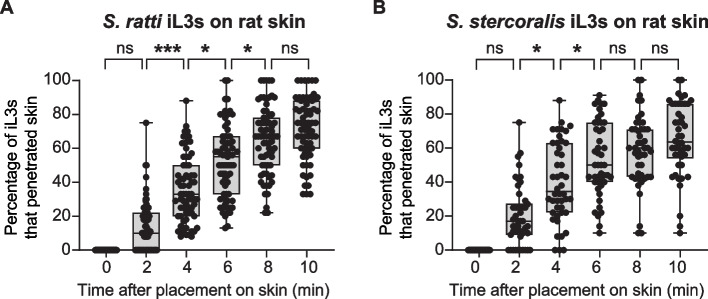
Both *S.* *ratti* and *S.* *stercoralis* progressively penetrate rat skin. **A**, **B** *S.* *ratti* iL3s (**A**) and *S.* *stercoralis* iL3s (**B**) penetrate rat skin over the course of 10 min in a population ex vivo skin-penetration assay. Box-and-whisker plots show the percentage of iL3s placed on the skin that had fully penetrated into the skin at the indicated time points. In the graphs, the boxes indicate the medians and interquartile ranges, and the error bars indicate the maximum and minimum values. *p < 0.05, ***p < 0.001, ns = not significant, Friedman test with Dunn’s post-test. *n* = 71 (A) or 46 (**B**) trials, with 7–12 iL3s per trial

**Fig. 2:**
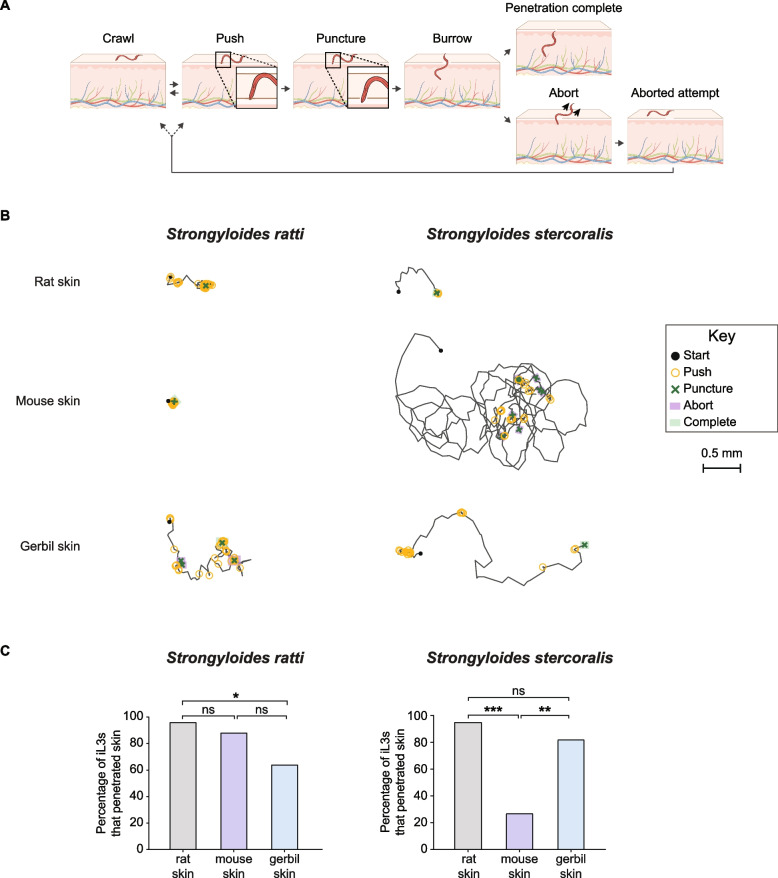
S. *ratti* and *S.* *stercoralis* iL3s penetrate different types of rodent skin with varying efficiencies. **A** Schematic illustrating the behavioral events executed by iL3s on the skin surface, as previously described [13]. After encountering skin, iL3s either crawl on the skin or push against the skin with their heads. A push sometimes results in a puncture, in which the head breaches the skin surface. The iL3 then proceeds to burrow into the skin. Some instances of burrowing result in the completion of penetration, where the entire body of the iL3 enters the skin. Other instances lead to aborted penetration attempts, in which the iL3 returns to the skin surface. Schematic is from Patel et al. [13]. **B** Behavioral tracks of representative *S. ratti* and *S. stercoralis* iL3s on the surface of rat, mouse, and gerbil skin from single-worm ex vivo skin-penetration assays. Black circles show the starting position of the iL3, and black lines show the trajectories of the iL3. Behavioral events are indicated in the key to the right. A worm was considered representative if the time it spent pushing and puncturing skin and the time to first puncture were close to the median value of the entire cohort, as previously described [13]. **C** The percentage of iL3s that complete penetration varies based on the iL3 species and the skin type. *S.* *ratti* more often completes penetration on rat skin than gerbil skin but completes penetration at similar frequencies on rat and mouse skin (left graph). In contrast, *S.* *stercoralis* more often completes penetration on rat and gerbil skin than mouse skin (right graph). *p < 0.05, **p < 0.01, ***p < 0.001, ns = not significant, Fisher’s exact test with Bonferroni correction for multiple comparisons. n = 24–25 *S.* *ratti* iL3s per skin type and 22 *S.* *stercoralis* iL3s per skin type. Note that there are no error bars because the graphs show the percentage of iL3s that completed penetration on each skin type out of the total iL3s tested

**Fig. 3:**
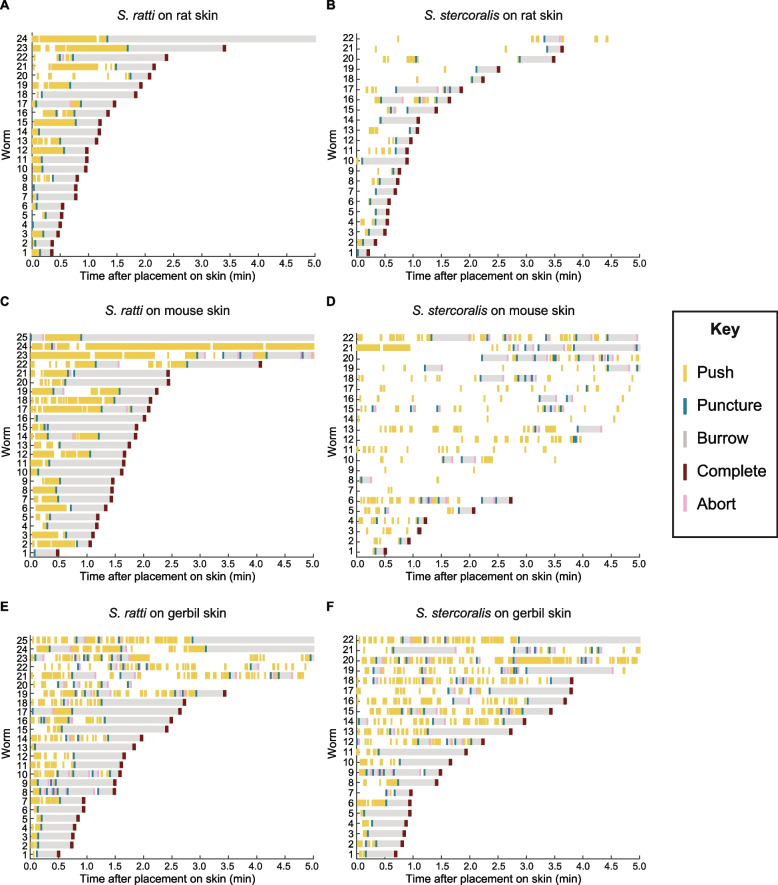
S. *ratti* and *S.* *stercoralis* iL3s spend varying amounts of time engaging in skin-penetration behavior on different types of rodent skin. Raster plots show the frequency and timing of the behavioral events defined in Fig. 2A for *S.* *ratti* on rat skin (**A**), *S.* *stercoralis* on rat skin (**B**), *S.* *ratti* on mouse skin (**C**), *S.* *stercoralis* on mouse skin (**D**), *S.* *ratti* on gerbil skin (**E**), and *S.* *stercoralis* on gerbil skin (**F**). The x-axes display the time after placement on the skin surface; the y-axes show individual iL3s that were tracked. Behavioral events are color-coded according to the key on the right. In each raster plot, worms are ordered based on the time at which they completed penetration

**Fig. 4:**
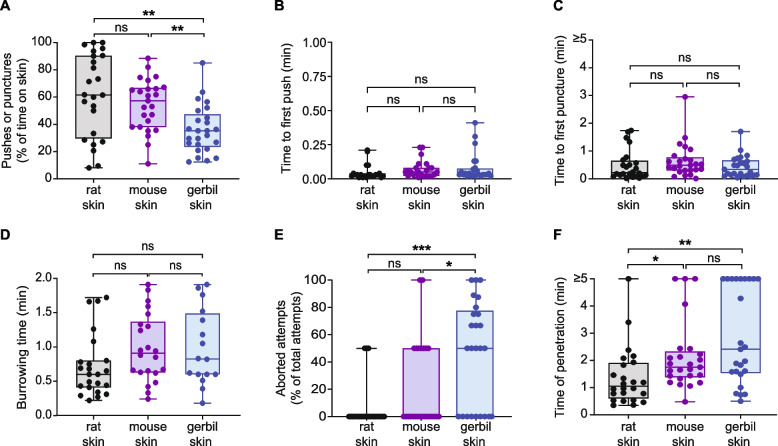
S. *ratti* shows an increased frequency of skin-penetration behavior on rat and mouse skin relative to gerbil skin. **A** *S.* *ratti* spends more time pushing and puncturing rat and mouse skin relative to gerbil skin. Graph shows the percentage of total time on skin spent pushing and puncturing each skin type. **B** *S.* *ratti* first pushes on rat, mouse, and gerbil skin at similar times after placement on the skin. **C** *S.* *ratti* first punctures rat, mouse, and gerbil skin at similar times after placement on the skin. **D** *S.* *ratti* spends a similar amount of time burrowing into rat, mouse, and gerbil skin. Burrowing time was defined as the time from the last puncture to the completion of penetration. **E** *S.* *ratti* more frequently aborts penetration attempts on gerbil skin relative to rat and mouse skin. Aborted penetration attempts are defined as in Fig. 2A. **F** *S.* *ratti* takes longer to penetrate gerbil and mouse skin than rat skin. Time of penetration was defined as the time from placement on the skin surface to the completion of penetration. For all graphs, circles represent values for individual worms, lines show medians and interquartile ranges, and error bars indicate ranges. *p < 0.05, **p < 0.01, ***p < 0.001, ns = not significant, one-way ANOVA with Dunnett’s post-test (**A**) or Kruskal–Wallis test with Dunn’s post-test (**B**-**F**). Data are quantifications of the behavioral events shown in Figs. 2 and 3. *n* = 16–23 worms per skin type. Behavioral parameters plotted were obtained from 5 independent biological replicates

**Fig. 5:**
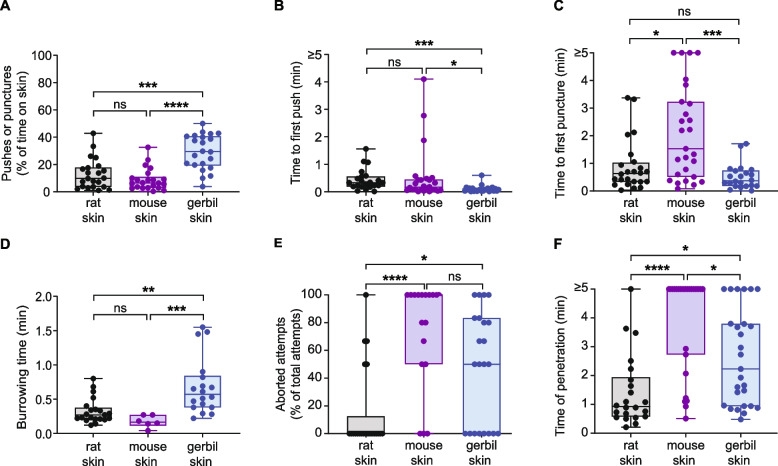
S. *stercoralis* engages in more skin-penetration behavior on gerbil skin relative to rat and mouse skin. **A** *S.* *stercoralis* spends more time pushing or puncturing gerbil skin than rat or mouse skin. Graph shows the percentage of time spent pushing and puncturing each skin type. **B** *S.* *stercoralis* first pushes on gerbil skin sooner than rat or mouse skin. **C** *S.* *stercoralis* first punctures rat and gerbil skin sooner than mouse skin. **D** *S.* *stercoralis* takes longer to burrow into gerbil skin than rat or mouse skin. Burrowing time was defined as in Fig. 4D. **E** *S.* *stercoralis* more frequently aborts penetration attempts on mouse and gerbil skin relative to rat skin. Aborted penetration attempts are defined as in Fig. 2A. **F** *S.* *stercoralis* penetrates rat skin most rapidly, followed by gerbil skin, followed by mouse skin. Time of penetration was defined as in Fig. 4F. For all graphs, circles represent values for individual worms, lines show medians and interquartile ranges, and error bars indicate ranges. *p < 0.05, **p < 0.01, ***p < 0.001, ****p < 0.0001, ns = not significant, Kruskal–Wallis test with Dunn’s post-test. Data are quantifications of the behavioral events shown in Figs. 2 and 3. *n* = 22–27 worms per skin type for all graphs except D; *n* = 6–21 worms per skin type for D because the graph only includes worms that completed penetration. Behavioral parameters plotted were obtained from 5 independent biological replicates

**Fig. 6:**
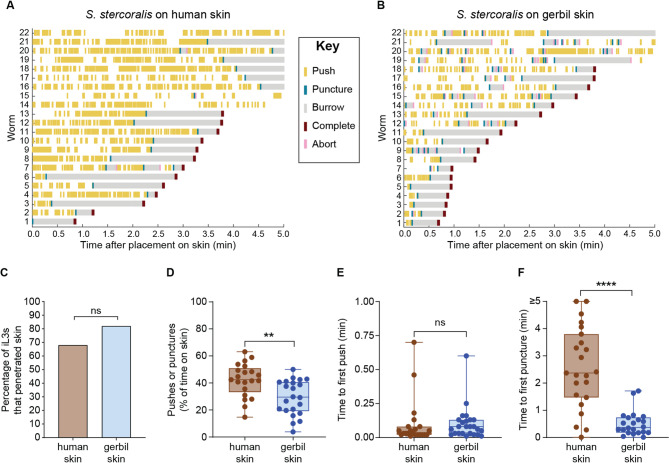
*S. stercoralis* engages in more skin-penetration behavior on human skin relative to gerbil skin. **A-B** *S. stercoralis* iL3s more robustly engage in skin-penetration behavior on human skin (**A**) relative to gerbil skin (**B**). Raster plots show the frequency and timing of the behavioral events defined in Fig. 2A. The x-axis displays the time after placement on the skin surface; the y-axis shows individual iL3s that were tracked. Behavioral events are color-coded according to the key in the center, and worms are ordered based on the time at which they completed penetration. **C** *S. stercoralis* iL3s penetrate human skin and gerbil skin at similar frequencies. ns = not significant, Fisher’s exact test. Note that there are no error bars because the graph shows the percentage of iL3s that completed penetration on human vs. gerbil skin out of the total iL3s tested. **D** *S. stercoralis* iL3s spend more time pushing and puncturing human skin than gerbil skin. **p<0.01, unpaired t-test. *n* = 20-22 iL3 per skin type. **E** *S. stercoralis* iL3s first push on human and gerbil skin at similar times. ns = not significant, Mann-Whitney test. *n* = 22-25 iL3s per skin type. **F** *S. stercoralis* iL3s first puncture gerbil skin sooner than human skin. ****p<0.0001, Mann-Whitney test. *n* = 22-23 iL3s per skin type. Data for gerbil skin are from Fig. 2, 3 and 5; data for human skin are from Patel et al. [13]

The following *S.* *stercoralis* strains were used: UPD (wild type) and EAH435 *bruIs4*[*Sst-act-2p::strmScarlet-I::Sst-era-1* 3′ UTR] [38]. The following *S.* *ratti* strains were used: ED231 (wild type) and EAH504 *bruIs15*[*Sst-act-2p::strmScarlet-I::Sst-era-1* 3′ UTR]; the transgenic red-fluorescent *S. ratti* strain is similar to the one that was previously described and was generated using the same methodology [38]. The wild-type *S. stercoralis* and *S. ratti* strains were used for conducting the studies shown in Fig. 1 and Fig. S1-S3 because, at this stage of the study, transgenic red-fluorescent *S. stercoralis* and *S. ratti* had not been generated yet. For these studies, wild-type *S.* *stercoralis* and *S. ratti* strains were stained with a fluorescent dye, DiI (see below for details on dye staining), in order to visualize the translucent iL3s on mammalian skin. The transgenic strains of *S.* *stercoralis* and *S.* *ratti* were used to generate data for Figs. 2, 3, 4, 5 and 6 because they express the fluorophore mScarlet-I throughout the body-wall muscle, which enables visualization of the iL3s on skin without the need to stain the iL3s with DiI [13, 38]. Skin-penetration behavior is similar in the transgenic iL3s vs. wild-type iL3s stained with dye [13]. Note that both the wild-type and transgenic strains of *S. stercoralis* and *S. ratti* used in this study are laboratory-adapted strains.

### Culturing and maintenance of *S. ratti*

Culturing and maintenance of *S.* *ratti* was performed as described [13]. Briefly, *S.* *ratti* was maintained by serial passage in Sprague–Dawley rats (*Rattus norvegicus *Hsd:Sprague Dawley SD, Inotiv) and cultured outside the rat on fecal-charcoal plates [13]. To infect rats*, S.* *ratti* iL3s were isolated from fecal-charcoal plates using a Baermann apparatus [39], washed five times in sterile 1 × PBS, and then injected subcutaneously into a rat anesthetized with isoflurane at a dose of ~ 700 iL3s in 200 µL sterile PBS. Typically, 2–4 rats were infected at a time for strain maintenance. Feces were collected from days 7–21 post-inoculation by housing rats on wire cage bottoms overnight, with damp cardboard (Shepherd Techboard, 8 × 16.5 inches, Newco, 999589) on the cage bottom. The following morning, feces were collected from the cage bottom and mixed with dH_2_O and autoclaved charcoal granules (Bone char, 4 lb. pail, 10 × 28 mesh, Ebonex). The fecal-charcoal mixture was then incubated in 10 cm Petri dishes (VWR, 82050–918) lined with damp Whatman paper; these fecal-charcoal plates were kept in plastic boxes lined with damp paper towels for moisture retention. The boxes containing the fecal-charcoal plates were stored either directly in a 23°C incubator or in a 20°C incubator for two days before storing at 23°C. For most *S. ratti* assays, iL3s were collected from plates that were 7–11 days old. To examine how the timing of the infection cycle affects skin penetration, 7–11-day-old plates made from feces collected on either days 7–10 (early in patency) or days 18–21 (late in patency) of the infection were used.

### Culturing and maintenance of *S. stercoralis*

Culturing and maintenance of *S.* *stercoralis* was performed as described [13]. Briefly, *S.* *stercoralis* was maintained by serial passage in Mongolian gerbils (*Meriones unguiculatus* Strain 243, Charles River Laboratories) and cultured outside the gerbil on fecal-charcoal plates [13]. To infect gerbils*, S.* *stercoralis* iL3s were isolated from fecal-charcoal plates using a Baermann apparatus [39], washed five times in sterile 1 × PBS, and then injected subcutaneously into a gerbil anesthetized with isoflurane at a dose of ~ 2,000 iL3s in 200 µL sterile PBS. Typically, 8–12 gerbils were infected at a time for strain maintenance. Feces were collected from days 14–44 post-inoculation according to the methods described above for *S.* *ratti*. For most *S.* *stercoralis* assays, iL3s were collected from plates that were 7–11 days old. To examine how the timing of the infection cycle affects skin penetration, 7–11-day-old plates made from feces collected on either days 14–23 (early in patency) or days 35–44 (late in patency) of the infection were used.

### Preparation of skin samples for ex vivo skin-penetration assays

For population ex vivo skin-penetration assays (Additional file 1: Fig. S1A) with rat skin, skin was obtained from male Sprague–Dawley rats (*Rattus norvegicus *Hsd:Sprague Dawley SD, Inotiv) aged 3–6 months. For assays examining how sex of the host affects skin penetration, skin from male and female Sprague–Dawley rats (*Rattus norvegicus *Hsd:Sprague Dawley SD, Inotiv) aged 3–6 months was used. For assays examining how host genetic background affects skin penetration, skin from female Sprague-Dawley rats (*Rattus norvegicus *Hsd:Sprague Dawley SD, Inotiv) aged 7-8 weeks and skin from male Wistar rats (*Rattus norvegicus *Hsd:WI Wistar, Inotiv) aged 7-8 weeks was used. For assays with gerbil skin, skin was obtained from male Mongolian gerbils (*Meriones unguiculatus *Strain 243, Charles River Laboratories) aged 2–4 months. For assays with mouse skin, skin was obtained from male C57BL/6 mice aged 2–4 months. Skin (from the epidermis to the hypodermis) was removed primarily from the dorsal region, although some skin explants were taken from the ventral region for rat and mouse assays. The skin was then either shaved using an electric razor or plucked to remove the fur. For population assays, the excised skin samples were stored at 4°C in a 10 cm Petri dish (VWR, 25384–342) that contained cotton squares (one + other, CVS pharmacy) soaked in BU saline [40]; 8–10 cotton squares were placed into each Petri dish. Skin explants were placed on top of the cotton in each Petri dish, and the dish was sealed with Parafilm™ (Bemis) and stored at 4°C until use. On each experimental day, the cotton and BU were replaced and only the skin being used that day was removed; the rest was stored at 4°C. Skin explants were stored at 4°C for up to ~ 5 days; we did not observe differences in skin-penetration behavior on skin explants that were stored at 4°C for up to 5 days (Additional file 1: Fig. S1B).

For single-worm ex vivo skin-penetration assays (Additional file 1: Fig. S1C) with rat skin, skin was prepared as previously described [13]. Briefly, rat skin was obtained from male Sprague–Dawley rats (*Rattus norvegicus *Hsd:Sprague Dawley SD, Inotiv) aged 8–11 months. Mouse skin was obtained from male C57BL/6 mice of varying ages, and gerbil skin was obtained from male Mongolian gerbils (*Meriones unguiculatus *Strain 243, Charles River Laboratories) aged 1.5–3 months. Skin from euthanized animals was removed as described above and then stored at −80°C until use. Similar morphology and permeability have been seen with fresh vs. frozen skin samples [41], and consistent with this, negligible differences in skin-penetration behavior were observed on fresh vs. frozen skin samples [13]. Before use, the samples were thawed and equilibrated to room temperature, and the fur was removed by manual plucking. Skin explants were then placed on top of BU-soaked cotton squares, as described above. For some assays, the skin was air-dried for 1–2 min prior to the start of the assay to remove excess moisture from the skin. The surface of the skin was lightly blotted with a BU-soaked cotton square between recording individual worms, to prevent the skin from drying out during the course of the assay. 

### Population ex vivo skin-penetration assays

Population ex vivo skin-penetration assays (Fig. 1A) were performed in 6-well plates (Corning, 3516). Small cotton squares were cut into 6 pieces, each approximately 2.5 cm × 2 cm. Skin explants were cut into smaller samples that were approximately 0.5 cm × 0.5 cm and then waxed with facial wax strips (Nad’s Facial Wax Strips, Item model #0790EN24, purchased from Amazon.com) to remove any remaining fur. The skin samples were then sandwiched between cotton squares saturated with BU saline [40] to retain moisture; the cotton on top of the skin was only removed immediately prior to the assay. Skin samples were placed into 2–3 wells of the plate, and additional BU was added to the empty wells and to the “moat” section of the plate to further aid in moisture retention. The plate was incubated at room temperature for 10–20 min prior to the start of the assay.

Infective larvae were obtained using a Baermann apparatus and then stained with DiI as previously described [13]. Briefly, a ~150 µL worm pellet was washed with BU saline and then resuspended in 10 mL BU saline. 1 mL of worm solution was pipetted into a watch glass, to which 5 µL of 2 mg/mL DiI (1,1'-dioctadecyl-3,3,3',3'-tetramethylindocarbocyanine perchlorate, Thermo Fisher Scientific, D3911) in N,N-dimethylformamide (Thermo Fisher Scientific, 68–12-2) was added. Worms were incubated with the DiI solution for 15–30 min. The dyed worms were then pipetted onto a plate containing Nematode Growth Medium (NGM) [42], and then transferred from the NGM plate onto the skin using a paintbrush (nail liner brush, Symphony, UPC #053742756612, purchased from Amazon.com). 7–12 worms were transferred onto each piece of skin and observed under a Leica M165 FC fluorescence dissection microscope with an ET-mCherry filter set (Leica, 10450195) for 10 min or until all iL3s had penetrated the skin. A new batch of DiI-stained worms was prepared every 30–45 min, and 0.5–1 mL BU saline was pipetted onto the sides of the cotton squares every time a new batch of dyed worms was prepared to retain moisture in the skin.

### Single-worm ex vivo skin-penetration assays

Single-worm ex vivo skin-penetration assays (Fig. S1C) were performed as previously described [13]. Briefly, *S.* *stercoralis* strain EAH435 and *S. ratti* strain EAH504 were used for assays because these transgenic worms contain an *Sst-act-2p::strmScarlet-I* transgene that labels body-wall muscle, thereby allowing visualization of the iL3s on the skin surface without the need for DiI staining [38]. The iL3s were pipetted onto an NGM plate, and an individual worm was then transferred from the plate onto the skin surface using a paintbrush. The worm was recorded using a Leica M165 FC fluorescence dissection microscope with an ET-mCherry filter set (Leica, 10450195) and an attached Basler ace (acA3800-14um) camera for 5 min or until the worm had fully penetrated into the skin or crawled off the skin surface. Still images were acquired at 4 frames/s using pylon Viewer software (Basler). The same skin sample was used for no more than 10 iL3s. To analyze worm behavior post hoc, every other image was opened in Fiji 2.9.0/1.53t [43] using File > Import > Image Sequence. Behaviors were scored manually, as previously described [13]. Pushing bouts were defined as the first frame in which the worm pushed on the skin until the frame prior to the one where the worm either resumed crawling or punctured the skin. To generate worm tracks, worm position was tracked in Fiji using the TrackMate plugin. Worm position was then plotted using previously published custom MATLAB software (https://github.com/BryantLabUW/WormTracker3000.git) [13].

### Statistical analysis

Statistical analysis was performed in Prism 10.4.2. The statistical tests used for each experiment are described in the figure legends. Two-tailed tests were used in all cases. Non-parametric tests were used when the data were non-parametric, as determined by tests for normality in Prism. Raster plots were generated using a custom MATLAB script, written with the help of Claude (https://claude.ai/new). The script imports the following parameters from an Excel file: 1) the frames that the iL3 started and finished pushing during each pushing bout; 2) each frame that the iL3 punctured the skin; 3) the frames that the iL3 started and finished burrowing during a penetration attempt; and 4) each frame that the iL3 either aborted or completed a penetration attempt. For behaviors wherein frame numbers are imported as ranges (pushing and burrowing), the script calculates all frames between the initial frame and final frame of each bout, in increments of 1, and stores this information. Next, all the frame numbers, both imported into and calculated within the script, are converted into minutes and plotted as a raster plot. The MATLAB script is available from GitHub (https://github.com/HallemLab/Abell_et_al_2025).

### Data availability

All raw data, results of statistical analyses, and original code are available from GitHub (https://github.com/HallemLab/Abell_et_al_2025). All raw images used in this study are available from BioImage Archive (https://www.ebi.ac.uk/biostudies/bioimages/studies/S-BIAD2189).

## Results

### Both *S. ratti* and *S. stercoralis* iL3s progressively penetrate rat skin

To investigate the ability of skin-penetrating iL3s to penetrate host vs. non-host skin, we first used population ex vivo skin-penetration assays in which 7–10 iL3s pre-soaked in the fluorescent dye DiI, which stains the nematode cuticle [44] and allows visualization on the skin surface [13], were placed onto excised, epilated rat skin. The number of iL3s visible on the skin surface was scored immediately and then repeatedly thereafter, at 2-min intervals for up to 10 min, using a fluorescence dissecting microscope (Additional file 1: Fig. S1A-B). Using this assay, we compared the ability of *S.* *ratti* and *S.* *stercoralis* iL3s to penetrate rat skin. We found that both *S.* *ratti* and *S.* *stercoralis* iL3s progressively penetrated rat skin over the course of 10 min (Fig. 1A-B). Some iL3s penetrated the skin within 2 min of placement on the skin, while others took longer to penetrate (Fig. 1A-B). Thus, both *S.* *ratti* and *S.* *stercoralis* iL3s are capable of penetrating rat skin.

### *S. ratti* and *S. stercoralis* iL3s behave differently on different rodent skin types

To gain insight into the behavior of iL3s on host vs. non-host skin, we conducted single-worm ex vivo skin-penetration assays in which the behavior of individual iL3s on the skin surface was video-recorded for up to 5 min and then analyzed post hoc, as previously described [13] (Additional file 1: Fig. S1C). For these assays, we used transgenic lines of *S.* *ratti* and *S.* *stercoralis* that stably express an *Sst-act-2p::strmScarlet-I* transgene in the body-wall muscle to enable visualization of the iL3s on the skin surface without the need for DiI [13, 38]. Behaviors on the skin were defined as pushes, in which the iL3 pushes its head down on the skin surface; punctures, in which the head of the iL3 breaches the skin surface; burrowing, in which the iL3 is partially inside the skin; completion of penetration, in which the entire body of the iL3 disappears below the skin surface; and aborted penetration, in which an iL3 that was partially inside the skin re-emerges such that its entire body is again on the skin surface (Fig. 2A).

Using this assay, we compared the behaviors of *S.* *ratti* and *S.* *stercoralis* iL3s on rat, mouse, and gerbil skin. We found that iL3s exhibit similar sequences of behavioral events on host vs. non-host skin (Figs. 2 and 3). In all cases, the iL3s engaged in repeated cycles of pushing, puncturing, and aborting on all three types of rodent skin (Figs. 2B and 3). However, the frequency and timing of these behaviors, and the proportion of iL3s that successfully completed penetration, differed depending on the skin type. For example, more *S.* *ratti* iL3s penetrated rat skin than gerbil skin (Fig. 2C). Additionally, *S. ratti* iL3s tended to immediately and continuously push down on rat and mouse skin (Figs. 2B and 3A, C), whereas the pushing bouts were more interrupted on gerbil skin (Figs. 2B and 3E). In contrast, more *S.* *stercoralis* iL3s penetrated rat and gerbil skin than mouse skin (Fig. 2C). *S. stercoralis* iL3s also pushed more frequently on the surface of gerbil skin than rat or mouse skin (Figs. 2B and 3B, D, F). For both *S. ratti *and *S. stercoralis, *many iL3s that did not successfully penetrate the skin nevertheless repeatedly engaged in skin-penetration behavior, sometimes nearly continuously, for the entire duration of the assay (Fig. 3). Together, these results suggest that the frequency and timing of skin-penetration behavior varies depending on the skin type.

### *S. ratti* iL3s penetrate host rat skin most efficiently

We then quantified these behavioral differences across the three skin types. In the case of *S.* *ratti*, we found that the iL3s spent more of their time on the skin surface pushing and puncturing rat and mouse skin than gerbil skin (Fig. 4A). Nevertheless, the time to first push and time to first puncture were similar across the three skin types (Fig. 4B-C). The burrowing time, defined as the time from the last puncture to the completion of penetration, was also similar on rat, mouse, and gerbil skin (Fig. 4D). However, *S.* *ratti* iL3s aborted penetration more frequently on gerbil skin relative to rat and mouse skin (Fig. 4E). Finally, the time from placement on the skin to the completion of penetration was lowest on rat skin and higher on mouse and gerbil skin (Fig. 4F). Taken together, these results suggest that *S.* *ratti* behaves differently on the skin of all three species of rodents and penetrates host rat skin most efficiently.

### *S. stercoralis* iL3s show increased skin-penetration behavior on gerbil skin but penetrate rat skin most efficiently

In the case of *S.* *stercoralis*, we found that the iL3s spent more time pushing and puncturing gerbil skin than rat or mouse skin (Fig. 5A). In addition, they began to push on gerbil skin sooner than on rat or mouse skin (Fig. 5B), and they took longer to puncture mouse skin than rat or gerbil skin (Fig. 5C). In contrast to *S.* *ratti*, which burrowed into all three rodent skin types with equal efficiency (Fig. 4D), *S.* *stercoralis* took longer to burrow into gerbil skin than rat or mouse skin (Fig. 5D), suggesting that gerbil skin may be more difficult for *S.* *stercoralis* iL3s to penetrate. *S.* *stercoralis* iL3s also aborted penetration attempts more often on mouse and gerbil skin than rat skin (Fig. 5E). Overall, the time from placement on the skin surface to the completion of penetration varied across skin types: the iL3s penetrated rat skin more rapidly than mouse or gerbil skin, and gerbil skin more rapidly than mouse skin (Fig. 5F). Together, these results suggest that *S.* *stercoralis* iL3s behave differently on all three rodent skin types, attempt penetration more frequently on some rodent skin types than others, and more efficiently penetrate some rodent skin types than others.

### *S. stercoralis* iL3s show increased skin-penetration behavior on human skin relative to gerbil skin

To investigate how *S.* *stercoralis* behaves on host vs. non-host skin, we compared the behavior of *S.* *stercoralis* iL3s on gerbil and human skin; human skin data, obtained using skin from the abdomen or breast of surgical patients following plastic surgery, were previously published [13]. We found that *S.* *stercoralis* iL3s showed an increased frequency of skin-penetration behavior on human skin relative to gerbil skin (Fig. 6A, B). Although the iL3s penetrated human and gerbil skin with similar frequency (Fig. 6C), they spent more time pushing/puncturing human skin than gerbil skin (Fig. 6D). In addition, although the iL3s began to push on human and gerbil skin at similar times (Fig. 6E), they took longer to puncture human skin than gerbil skin (Fig. 6F), suggesting that human skin is more difficult to penetrate than gerbil skin. Together, these results suggest that *S.* *stercoralis* iL3s show an increased frequency of skin-penetration behavior on human (host) skin relative to gerbil (non-host) skin, even though human skin is more difficult to penetrate.

### Skin-penetration behavior is robust to differences in age, sex, and genetic background of the skin donor

Finally, we asked whether skin-penetration ability varies depending on different parasite and host factors such as when during the course of the infection the iL3s were generated as well as the age, sex, and genetic background of the host. Using the population ex vivo skin-penetration assay (Additional file 1: Fig. S1A), we first compared skin-penetration behavior on rat skin in iL3s that were generated early vs. late in the course of the infection. In the case of rats experimentally infected with *S.* *ratti*, the infection lasts from days ~7–21 post-infection, after which the rat immune system clears the infection. In the case of gerbils experimentally infected with *S.* *stercoralis*, the infection lasts from days ~14–44 post-infection. We found that for both *S.* *ratti* and *S.* *stercoralis*, iL3s obtained from early vs. late in the infection were equally efficient at penetrating rat skin (Additional file 2: Fig. S2). Thus, even iL3s that are generated when the host has mounted a robust immune response to the infection can efficiently penetrate skin. We then compared the ability of *S.* *ratti* iL3s to penetrate skin from young (3-month-old) vs. older (8-month-old) rats, male vs. female rats, and Wistar vs. Sprague–Dawley rats. In all cases, we found that the iL3s penetrated rat skin with similar efficiency (Additional file 3: Fig. S3). These results suggest that skin-penetration behavior is robust to differences in skin age, sex, and genetic background, although we cannot exclude the possibility that it varies depending on factors not tested here.

## Discussion

Skin-penetrating nematodes in the genera *Necator, Ancylostoma,* and *Strongyloides* cause extensive morbidity and mortality worldwide [1, 45, 46]. *S.* *stercoralis* infections are of particular concern because infections can be chronic due to the unique autoinfective life cycle of this species, which enables the nematodes to cycle through multiple generations inside the same host [9]. In addition, infections are often fatal for immunosuppressed individuals [9]. Although anthelmintic drugs such as ivermectin can clear existing infections, the drugs do not prevent reinfection and treatment failure is not uncommon [47–49]. Moreover, drug resistance has been reported in *Strongyloides* species that infect ruminants [50] and has been observed in *S.* *ratti* following repeated exposure to anthelmintic drugs [11], raising the possibility that it will arise in *S.* *stercoralis* in the near future. Skin penetration is a critical step of the infection process, and as such, it presents a possible opportunity for anthelmintic intervention: topical compounds that block skin penetration could be used to prevent infections from establishing. However, the process of skin penetration remains poorly understood. Here, we conducted an in-depth analysis of *S.* *stercoralis* and *S.* *ratti* skin-penetration behavior on different skin types and showed that the timing and frequency of these behaviors varies depending on the skin type. Our results suggest that a better understanding of the skin properties and surface features that modulate skin-penetration behavior could inform the development of topical anthelmintics.

Overall, we find that both *S.* *ratti* and *S.* *stercoralis* iL3s behave differently on the three different types of rodent skin (Figs. 2, 3, 4 and 5). In the case of *S.* *ratti,* iL3s spend more time pushing and puncturing rat and mouse skin than gerbil skin (Fig. 4A) but push down at roughly the same time on all three skin types (Fig. 4B). These observations indicate that iL3s are quick to attempt penetration on all three skin types, but then repeatedly exhibit skin-penetration behavior on rat and mouse skin. Ultimately, more *S. ratti* iL3s penetrate rat skin than gerbil skin (Fig. 2C). Additionally, the iL3s penetrate rat skin more rapidly than mouse or gerbil skin (Fig. 4F). Together, these results suggest that *S.* *ratti* is more efficient at penetrating rat (host) skin than gerbil and mouse (non-host) skin. However, the behavior of *S.* *ratti* iL3s on rat and mouse skin is similar overall (Fig. 4), consistent with the close genetic relatedness of rats and mice [51, 52]. Interestingly, the observation that *S.* *ratti* more often aborts penetration attempts on gerbil skin than rat or mouse skin (Fig. 4E) suggests that skin penetration, and by extension, entry of the parasite into the body of an animal, is regulated both at the level of pushes/punctures and at the level of aborted penetration attempts.

In the case of *S.* *stercoralis*, a comparison of iL3 behavior on gerbil vs. mouse skin revealed that iL3s spend more time pushing/puncturing and push down more quickly on gerbil skin than mouse skin (Figs. 2B and 5A-B). They also take longer to puncture mouse skin than gerbil skin (Fig. 5C). Together, these results are consistent with an increased penetration drive on gerbil skin relative to mouse skin. Interestingly, *S.* *stercoralis* iL3s take longer to burrow into gerbil skin than mouse skin (Fig. 5D), suggesting that gerbil skin is more difficult to penetrate. However, more *S. stercoralis* iL3s penetrated gerbil skin than mouse skin (Fig. 2C), indicating that an increased penetration drive may compensate for the increased difficulty of entering the skin. On human skin, *S.* *stercoralis* iL3s show even more frequent pushes and punctures than on gerbil skin (Fig. 6A, B, D); because humans are a definitive host for *S.* *stercoralis*, the increased penetration drive on this skin type might be stimulated by host-specific cues. However, human skin appears to be even tougher to penetrate than gerbil skin, since the first puncture occurs significantly later on human skin (Fig. 6F); this is consistent with the increased stiffness of human skin relative to rodent skin [53]. Nevertheless, the percentage of *S.* *stercoralis* iL3s that successfully penetrate human skin and gerbil skin are similar (Fig. 6C). These results further bolster the idea that increased penetration drive can enable skin penetration on skin types that are harder to penetrate. In addition to penetration drive, which is presumably influenced by the detection of skin surface features and other skin sensory cues by the parasite, penetration ability and efficiency across skin types also likely reflects differences in the biochemical and/or mechanical properties of the skin. Further investigations are needed to tease apart the relative contributions of parasite penetration drive vs. skin physical properties to skin penetration. In future studies, it will also be interesting to test whether skin-penetration behavior differs among *S.* *stercoralis* strains isolated from humans vs. dogs [23, 33–35] to determine whether skin-penetration behavior contributes to differences in parasite prevalence among different host populations.

The specific features of the skin that stimulate skin-penetration behavior remain unknown. The skin from different mammalian species differs in surface topography; thickness; mechanical properties such as elasticity and viscoelasticity; and surface features such as the density, size, and spacing of hair follicles [53–57]. Skin thickness and surface features also differ across individuals of the same species and locations on the body [53]. In addition, skin thickness, mechanical properties, and surface topography change dramatically as a function of age [58–61]. Skin surface topography, features, and mechanical properties likely all influence skin-penetration behavior. Consistent with this possibility, we recently showed that *S.* *stercoralis* requires signaling from dopaminergic neurons for skin penetration [13]; these neurons are presumed to be mechanosensory based on knowledge of homologous neurons in the free-living nematode *Caenorhabditis* *elegans* [62–66]. Moreover, the putative mechanosensory TRP channel gene *Sst-trp-4* is expressed in the *S. stercoralis* dopaminergic neurons and is required for skin penetration [13], further supporting the model that mechanosensation modulates skin penetration. Thus, the detection of skin surface properties by mechanosensory neurons is likely to be critical for driving skin penetration. In addition to mechanosensory cues, chemosensory cues vary greatly across species [67–71] and may also influence skin penetration.

In addition to understanding the specific skin features that stimulate skin-penetration behavior, it will be important to understand whether skin from host vs. non-host mammals elicits different physiological responses from iL3s. For example, host skin could stimulate more frequent or more robust muscle contractions in worms than non-host skin, thus enabling worms to better propel themselves into host skin. Additionally, differences in muscle composition, cuticle composition, or sensorimotor neural circuits between different parasitic nematode species could contribute to the increased ability of a specific nematode species to penetrate some skin types over others. For example, it is possible that the body-wall muscle of *S. stercoralis* generates more force than that of *S. ratti*, allowing *S. stercoralis* to burrow more effectively than *S. ratti* into skin types that are tougher and less pliable, such as human skin [53]. The ability to make stable transgenic and knockout lines of *S.* *stercoralis* and *S.* *ratti* [13, 15, 38] paves the way for future investigations into how species-specific differences in nematode biology and physiology contribute to differences in the interactions of iL3s with different skin types.

At a biochemical level, skin-penetrating iL3s are thought to use astacin metalloproteases to digest and burrow into skin [72]. Although a direct requirement for specific astacin metalloproteases in skin penetration has not yet been demonstrated, several lines of evidence point to an important role for astacins in skin penetration: the genomes of skin-penetrating iL3s contain large families of astacin metalloprotease genes, multiple astacins are secreted in excretory-secretory (ES) product, at least some astacins can digest skin collagen in vitro, and exposing iL3s to metalloprotease inhibitors blocks skin penetration [72–84]. It is possible that astacins differ in their substrate specificity and that individual astacins are more effective at digesting some skin types than others, thus contributing to the differing abilities of iL3s to penetrate into various types of skin. In addition, some mammals have softer and thinner skin than other mammals – for example, rat skin is softer and thinner than human skin [53] – and differences in skin softness and thickness, as well as differences in the relative amounts of astacins secreted by different species of skin-penetrating nematodes, may together contribute to the differing ability of these nematodes to digest and penetrate the skin of different mammalian species. The results presented here provide a foundation for future studies aimed at investigating possible relationships between the astacin repertoire of iL3s and skin penetration efficiency. In addition, the contributions of other host proteins, including other host proteases, to skin penetration is an important direction for future research.

Finally, in this study, skin penetration was studied using an ex vivo skin-penetration assay [13]. While this assay provides a foundation for understanding skin-penetration behavior, it is important to note that skin explants lack the temperature, oxygen, and carbon dioxide gradients found in intact skin [85–88]. In addition, the composition of lipids, sebum, and other skin chemicals may differ following skin removal [89–92]. Skin-penetrating iL3s exhibit robust behavioral responses to temperature, respiratory gases, and host-associated chemosensory cues [15, 17, 93]; responses to these sensory stimuli are likely to modulate skin-penetration behavior. Skin-penetration behavior is also likely influenced by the cutaneous immune response of the host during skin invasion [94]. Thus, in future studies it will be important to study skin penetration in vivo using live animals as well as ex vivo using skin explants.

## Conclusions

In conclusion, our results illustrate that the timing and frequency of skin-penetration behavior exhibited by iL3s varies depending on the skin type. Thus, the unique behavioral cycles executed by skin-penetrating iL3s and the composition of the skin barrier both appear to be important determinants of host specificity. Our results will inform future studies of the specific properties and features of skin that are detected by iL3s to stimulate skin penetration.

## Supplementary Information

Supplementary Material 1: Figure S1. An ex vivo skin penetration assay. A. Diagram of the population ex vivo skin-penetration assay. Skin from either rats, mice, or gerbils was excised from a euthanized animal and epilated. The skin was then used immediately or placed at 4°C for up to ~ 5 days prior to use. For assays, the skin samples were placed in a 6-well plate sandwiched between saline-soaked cotton pads for moisture retention. The cotton pad on top of the skin was removed immediately prior to the assay. For each assay, 7–12 iL3s, stained with DiI to allow visualization on the skin surface, were placed onto the skin and observed under a fluorescence dissecting microscope for 10 min. B. *S. ratti* iL3s penetrated rat skin with similar efficiency when the rat skin was used 0–1 days post-euthanasia or 4–5 days post-euthanasia. *p* = 0.8497, two-way repeated measures ANOVA. n = 91 trials for skin aged 0–1 days and 37 trials for skin aged 4–5 days, with 7–12 iL3s per trial. C. Diagram of the single-worm ex vivo skin-penetration assay. Skin was prepared as described above, except that it was frozen at -80°C prior to use; negligible differences in skin-penetration behavior were observed between fresh and frozen skin samples [13]. Skin samples were placed into a dish sandwiched between saline-soaked cotton pads for moisture retention, and the cotton pad on top of the skin was removed immediately prior to the assay. Individual iL3s expressing an integrated *Sst-act-2p::strmScarlet-I* transgene [13] were placed on the skin, and behavior was recorded for 5 min or until the iL3 penetrated the skin or crawled off the skin surface. The transgene consists of the *Sst-act-2* promoter, which drives expression in body-wall muscle, and a *Strongyloides*-codon-optimized *mScarlet-I* reporter gene [13]. Schematic is adapted from Patel et al. [13].

Supplementary Material 2: Figure S2. iL3s from early vs. late in the infection penetrate skin with similar efficiency. A. *S. ratti* iL3s from early in the infection penetrate at similar rates as *S.* *ratti* iL3s from late in the infection. *p* = 0.2004, two-way repeated measures ANOVA. *n* = 24–41 trials, with 7–12 iL3s per trial. B. *S. stercoralis* iL3s from early in the infection penetrate with similar efficiency to those from late in the infection. *p* = 0.6769, two-way repeated measures ANOVA. *n* = 13–16 trials per condition, with 7–12 iL3s per trial.

Supplementary Material 3: Figure S3. Skin donor age, sex, and genetic background do not affect skin penetration. A. *S.* *ratti* iL3s penetrate skin from a 3-month-old rat and skin from an 8-month-old rat with similar efficiency. *p* = 0.8430, two-way repeated measures ANOVA. *n* = 30–31 trials per condition, with 7–12 iL3s per trial. B. *S.* *ratti* iL3s penetrate skin from male and female rats with similar efficiency. *p* = 0.1313, two-way repeated measures ANOVA. *n* = 23–24 trials per condition, with 7–12 iL3s per trial. C. *S.* *ratti* iL3s penetrate skin from Sprague-Dawley and Wistar rats with similar efficiency. *p* = 0.9792, two-way repeated measures ANOVA. *n* = 16 trials per condition, with 7–12 iL3s per trial.

## Data Availability

All raw data, results of statistical analyses, and original code are available from GitHub (https://github.com/HallemLab/Abell_et_al_2025). All raw images used in this study are available from BioImage Archive (https://www.ebi.ac.uk/biostudies/bioimages/studies/S-BIAD2189).
