# Supplementary figures and images for: Strongyloides species exhibit distinct behaviors on the skin of different mammals
Source: BMC Infect Dis. 2025 Oct 3;25:1233. doi: 10.1186/s12879-025-11543-9 (PMC12495621; doi:10.1186/s12879-025-11543-9)

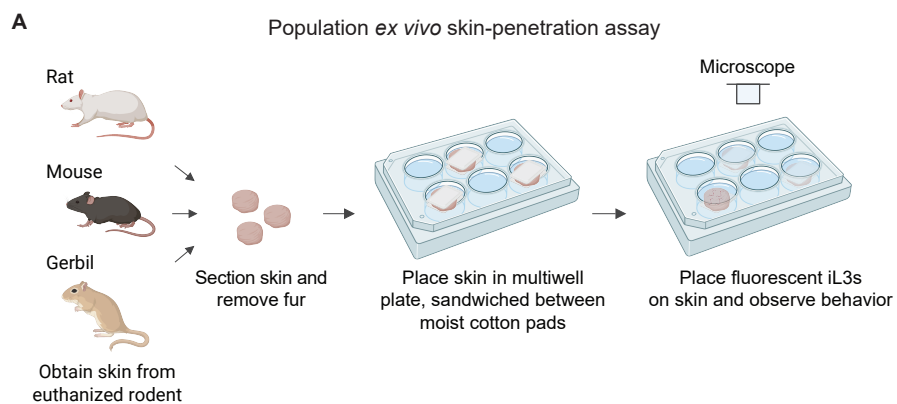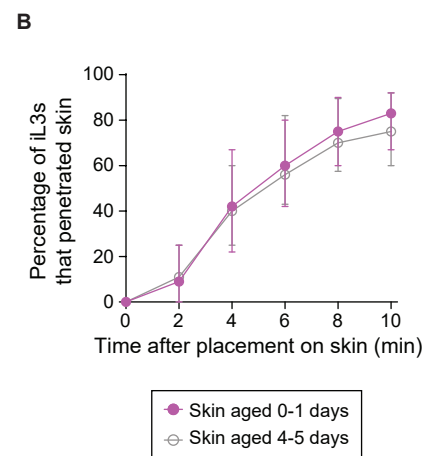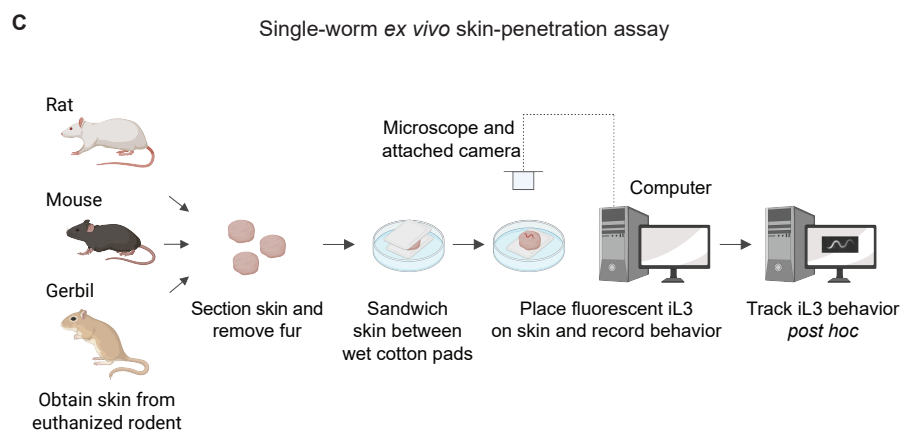

Supplement: Supplementary file 1 — Supplementary Material 1: Figure S1. An ex vivo skin penetration assay. A. Diagram of the population ex vivo skin-penetration assay. Skin from either rats, mice, or gerbils was excised from a euthanized animal and epilated. The skin was then used immediately or placed at 4°C for up to ~ 5 days prior to use. For assays, the skin samples were placed in a 6-well plate sandwiched between saline-soaked cotton pads for moisture retention. The cotton pad on top of the skin was removed immediately prior to the assay. For each assay, 7–12 iL3s, stained with DiI to allow visualization on the skin surface, were placed onto the skin and observed under a fluorescence dissecting microscope for 10 min. B. S. ratti iL3s penetrated rat skin with similar efficiency when the rat skin was used 0–1 days post-euthanasia or 4–5 days post-euthanasia. p = 0.8497, two-way repeated measures ANOVA. n = 91 trials for skin aged 0–1 days and 37 trials for skin aged 4–5 days, with 7–12 iL3s per trial. C. Diagram of the single-worm ex vivo skin-penetration assay. Skin was prepared as described above, except that it was frozen at -80°C prior to use; negligible differences in skin-penetration behavior were observed between fresh and frozen skin samples [13]. Skin samples were placed into a dish sandwiched between saline-soaked cotton pads for moisture retention, and the cotton pad on top of the skin was removed immediately prior to the assay. Individual iL3s expressing an integrated Sst-act-2p::strmScarlet-I transgene [13] were placed on the skin, and behavior was recorded for 5 min or until the iL3 penetrated the skin or crawled off the skin surface. The transgene consists of the Sst-act-2 promoter, which drives expression in body-wall muscle, and a Strongyloides-codon-optimized mScarlet-I reporter gene [13]. Schematic is adapted from Patel et al. [13]. [file 12879_2025_11543_MOESM1_ESM.pdf]

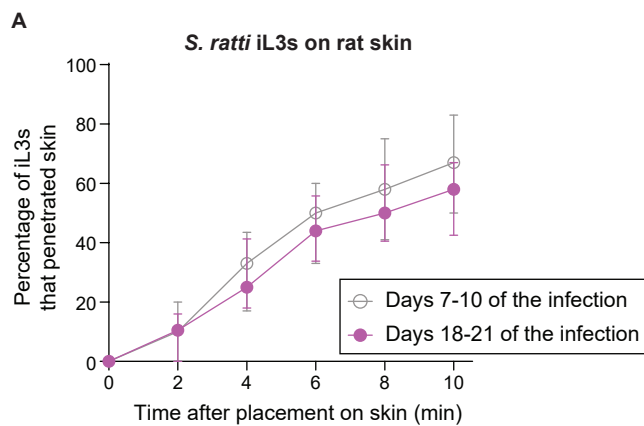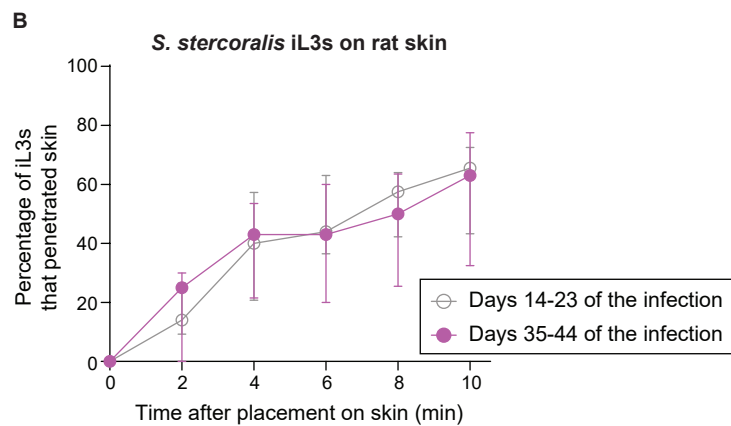

Supplement: Supplementary file 2 — Supplementary Material 2: Figure S2. iL3s from early vs. late in the infection penetrate skin with similar efficiency. A. S. ratti iL3s from early in the infection penetrate at similar rates as S. ratti iL3s from late in the infection. p = 0.2004, two-way repeated measures ANOVA. n = 24–41 trials, with 7–12 iL3s per trial. B. S. stercoralis iL3s from early in the infection penetrate with similar efficiency to those from late in the infection. p = 0.6769, two-way repeated measures ANOVA. n = 13–16 trials per condition, with 7–12 iL3s per trial. [file 12879_2025_11543_MOESM2_ESM.pdf]

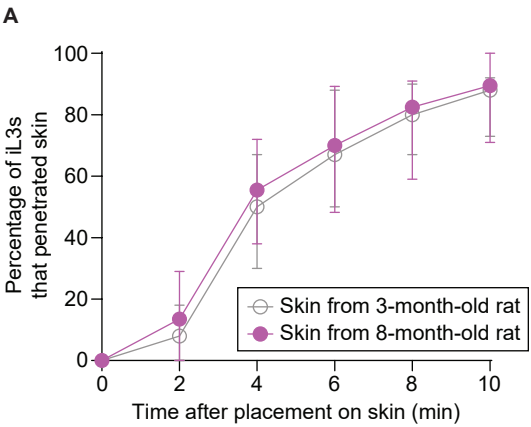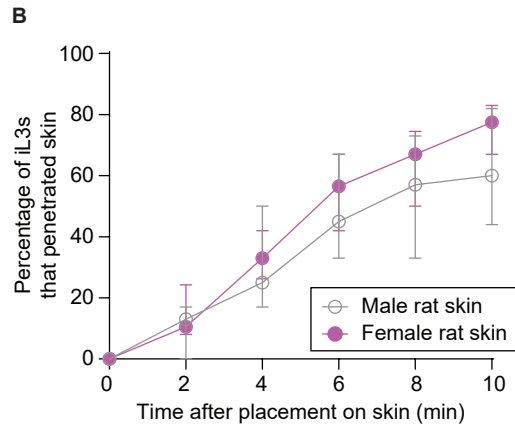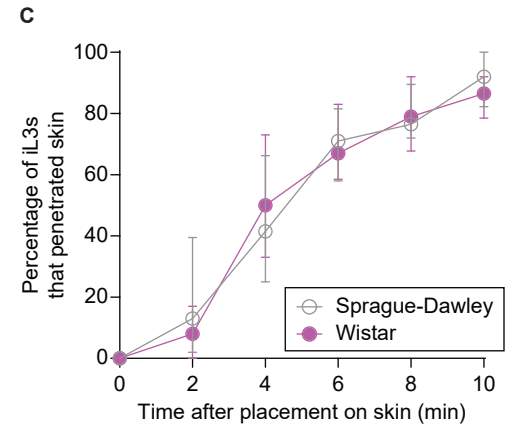

Supplement: Supplementary file 3 — Supplementary Material 3: Figure S3. Skin donor age, sex, and genetic background do not affect skin penetration. A. S. ratti iL3s penetrate skin from a 3-month-old rat and skin from an 8-month-old rat with similar efficiency. p = 0.8430, two-way repeated measures ANOVA. n = 30–31 trials per condition, with 7–12 iL3s per trial. B. S. ratti iL3s penetrate skin from male and female rats with similar efficiency. p = 0.1313, two-way repeated measures ANOVA. n = 23–24 trials per condition, with 7–12 iL3s per trial. C. S. ratti iL3s penetrate skin from Sprague-Dawley and Wistar rats with similar efficiency. p = 0.9792, two-way repeated measures ANOVA. n = 16 trials per condition, with 7–12 iL3s per trial. [file 12879_2025_11543_MOESM3_ESM.pdf]
